# Pathway from Acute Kidney Injury to Chronic Kidney Disease: Molecules Involved in Renal Fibrosis

**DOI:** 10.3390/ijms241814019

**Published:** 2023-09-13

**Authors:** Andrei Niculae, Mihai-Emil Gherghina, Ileana Peride, Mirela Tiglis, Ana-Maria Nechita, Ionel Alexandru Checherita

**Affiliations:** 1 Department of Nephrology, Clinical Department No. 3, “Carol Davila” University of Medicine and Pharmacy, 050474 Bucharest, Romania; niculaeandrei@yahoo.com; 2 Department of Nephrology, Ilfov County Emergency Clinical Hospital, 022104 Bucharest, Romania; 3 Department of Anesthesia and Intensive Care, Emergency Clinical Hospital of Bucharest, 014461 Bucharest, Romania; 4 Department of Nephrology, “St. John” Emergency Clinical Hospital, 042122 Bucharest, Romania; 5 Research Department, Emergency Clinical Hospital of Bucharest, 014461 Bucharest, Romania; al.checherita@gmail.com

**Keywords:** acute kidney injury, chronic kidney disease, renal fibrosis pathways, pro-fibrotic factors, treatment

## Abstract

Acute kidney injury (AKI) is one of the main conditions responsible for chronic kidney disease (CKD), including end-stage renal disease (ESRD) as a long-term complication. Besides short-term complications, such as electrolyte and acid-base disorders, fluid overload, bleeding complications or immune dysfunctions, AKI can develop chronic injuries and subsequent CKD through renal fibrosis pathways. Kidney fibrosis is a pathological process defined by excessive extracellular matrix (ECM) deposition, evidenced in chronic kidney injuries with maladaptive architecture restoration. So far, cited maladaptive kidney processes responsible for AKI to CKD transition were epithelial, endothelial, pericyte, macrophage and fibroblast transition to myofibroblasts. These are responsible for smooth muscle actin (SMA) synthesis and abnormal renal architecture. Recently, AKI progress to CKD or ESRD gained a lot of interest, with impressive progression in discovering the mechanisms involved in renal fibrosis, including cellular and molecular pathways. Risk factors mentioned in AKI progression to CKD are frequency and severity of kidney injury, chronic diseases such as uncontrolled hypertension, diabetes mellitus, obesity and unmodifiable risk factors (i.e., genetics, older age or gender). To provide a better understanding of AKI transition to CKD, we have selected relevant and updated information regarding the risk factors responsible for AKIs unfavorable long-term evolution and mechanisms incriminated in the progression to a chronic state, along with possible therapeutic approaches in preventing or delaying CKD from AKI.

## 1. Introduction

There is a bidirectional association between acute kidney injury (AKI) and chronic kidney disease (CKD). In other words, CKD can induce AKI, but AKI can induce CKD via pro-fibrotic signaling pathways as well. Not all AKI patients will experience CKD; therefore, it is important to identify those subjects with the greatest risk for renal fibrosis and, eventually, CKD or end-stage renal disease (ESRD). Takaori et al. sustain that the degree of fibrosis is driven by the nature of kidney injury, the extent of the tubular damage, the duration, severity and also frequency of the tubular lesion [1]. Gender as a risk factor is evidenced by Hewitson and others through their investigation, which suggests that males are more prone to CKD from AKI because of the damaging effects of testosterone rather than estrogen, which has no influence on tubular injury [2]. Recent findings in understanding the pathogenic pathway of renal fibrosis evidenced mechanisms responsible for normal and abnormal recovery injury. Thereby, a link between renal injury, abnormal repair and progression to fibrosis was established. Consequently, it was suggested that the key element involved in renal fibrosis is a maladaptive repair process [3]. The proposed maladaptive kidney regeneration events were epithelial tubule arrested growth, epithelial dedifferentiate dysfunction, tubular atrophy, mitochondrial dysfunction, pathological paracrine and autocrine activity, which perturb the normal interaction between tubular, epithelial, endothelial, fibroblast and inflammatory cells [3].

## 2. Epidemiology of AKI to CKD Transition

AKI is a syndrome defined by a serum creatinine (SCr) increase of more than 0.3 mg/dL over baseline SCr or a 1.5× baseline SCr increase within in the last 7 days or urinary volume under 0.5 mL/bwt/h in the last 6 h [4]. Between 2004 and 2012, a systematic review evidenced an AKI incidence of 21.6% in adults and 33.7% in pediatric hospital setting population [5]. Approximately 10% of AKI pediatric patients developed CKD stages 1–3, including 9% of patients with persistent proteinuria, 4.5% patients with AKI KDIGO (representing Kidney Disease Improving Global Outcomes) stage 1, 10.6% patients with AKI KDIGO stage 2 and 17.1% patients with AKI KDIGO stage 3 [6]. Ishani et al. reported that hospitalized AKI patients had a 6.74% probability of needing renal replacement therapy (RRT) vs. non-AKI patients during the 2-year follow-up. In addition, they demonstrated that 41.2% of hospitalized AKI patients with previous CKD developed ESRD [7]. Another recent study with a 3-year follow-up period showed that 24% of AKI-exposed patients developed CKD [8]. Therefore, AKI can be recognized as an important risk factor in CKD onset or acceleration of ESRD. Factors that may be implicated in AKIs progression into CKD include AKI severity, time till recovery, number of AKI episodes, pre-existent CKD and proteinuria persistence [9].

## 3. Pathway to Renal Fibrosis

**Figure 1:**
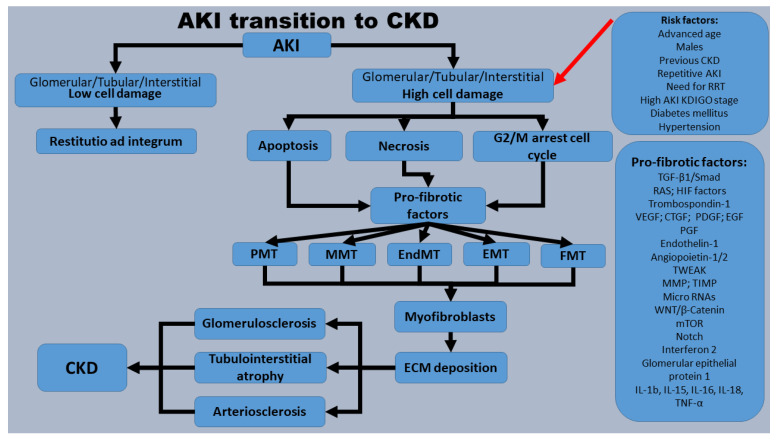
Pathogenic mechanisms involved in AKI transition to CKD. The mentioned risk factors prone AKI subjects to higher glomerular and/or tubule-interstitial system cellular damage. Furthermore, apoptotic, necrotic and senescent cells (cells blocked in the G2/M cell cycle) release and stimulate pro-fibrotic factors, which, via FMT, PMT, EMT, EndMT and MMT, will generate myofibroblasts. Myofibroblasts secrete ECM that will generate glomerulosclerosis, tubulointerstitial fibrosis and arteriosclerosis, defined as CKD. Acronyms: AKI—acute kidney injury; CKD—chronic kidney disease; CTGF—connective tissue growth factor; ECM—extracellular matrix; EGF—epidermal growth factor; EMT—epithelial to mesenchymal transition; EndMT—endothelial to mesenchymal transition; FMT—fibroblast to myofibroblast transition; HIF—hypoxia-inducible factor; KDIGO—Kidney Disease Improving Global Outcomes; IL—interleukin; MMP-7—matrix metalloproteinase-7; MMT—macrophage myofibroblast transition; mTOR—mammalian target of rapamycin; Notch—neurogenic locus notch homolog protein; PDGF—platelet-derived growth factor; PGF—placental growth factor; PMT—pericyte to myofibroblast-transition; RAS—renin-angiotensin system; RNAs—ribonucleic acids; TGF-β1—transforming growth factor; TIMP—tissue inhibitor of metalloproteinase; TNF-α—tumor necrosis factor alpha; TWEAK—TNF-like weak inducer of apoptosis; VEGF—vascular endothelial growth factor; Wnt/β-catenin—wingless/beta-catenin.

Apparently, myofibroblasts are the pathogenic key factors in extracellular matrix (ECM) synthesis, which is subsequently responsible for renal fibrosis. The origin of myofibroblasts is still undetermined, but some authors consider that resident pericytes, fibroblasts, epithelial, endothelial and bone marrow cells can be a potential source [10]. Therefore, in the pathogenic mechanism of renal fibrosis, we can take into consideration the next general concepts as key generators for myofibroblasts: fibroblast to myofibroblast transition (FMT) [11], pericyte to myofibroblast-transition (PMT) [12], epithelial to mesenchymal transition (EMT) [13], endothelial to mesenchymal transition (EndMT) [13], macrophage (bone-marrow-derived) myofibroblast transition (MMT) [14]. Transition to myofibroblasts from different potential progenitors can be recognized by smooth muscle actin (SMA) expression [15] (Figure 1).

## 4. Main Pro-Fibrotic Factors and Mechanisms of Action

Previous studies evidenced multiple molecules involved in pro-fibrotic pathways, many of them interconnected: Wnt/β-catenin, TGF-β/SMAD signaling, RAS system, transcriptional factors (Snail factor, MMP-7, plasminogen activator inhibitor-1, micro or long ribonucleic acids, TRPC-6, etc.), death cell signaling, ischemic, inflammation or vascular factors. The most important factors responsible for kidney fibrosis will be described further on.

### 4.1. Wnt/β-Catenin Signaling Pathway

Wingless (Wnt)/β-catenin is heavily involved in the development of renal fibrosis, being involved in the control of different pro-fibrotic pathways, such as transforming growth factor beta-1 (TGF-β1)/SMAD (family of proteins that are the main signal transducers for TGF beta receptors) signaling, renin-angiotensin system (RAS), Snail1 (representing the gene encoded a nuclear protein similar to Drosophila embryonic protein snail), twist-related protein 1 (Twist1), matrix metalloproteinase-7 (MMP-7), transient receptor potential canonical 6 (TRPC6), plasmin activator inhibitor-1 (PAI-1), fibroblasts and macrophages [16]. There are numerous studies that show a decreased renal fibrosis when this pathway is blocked [17]. Studies evidenced that Wnt/β-catenin is a key regulator of tissue organization in embryonic life, but it can be re-expressed in adult life in different diseases. As an example, in AKI, transient Wnt/β-catenin activation is responsible for repairs and cellular regeneration, whereas constant and uncontrolled secretion promotes CKD via renal fibrosis, mineral bone disease, podocyte damage and proteinuria [18]. Wnt is derived from tubular cells, and the signaling pathway process is divided into a canonical pathway named Wnt/β-catenin and a non-canonical line of action, also subdivided into Wnt/planar cell polarity (PCP) and Wnt/calcium (Ca^2+^) pathways [19]. The Wnt/β-catenin pathway starts into the cells’ cytoplasm. Under normal conditions, cytoplasmic β-catenin is inactivated by the “destruction complex”, which includes five proteins named Axin, casein kinase 1 (CK1), adenomatous polyposis coli (APC), glycogen synthase kinase 3β (GSK3β) and disheveled (DVL). This complex will induce β-catenin phosphorylation to become inactive. In pathological states, secreted Wnt ligands bind to Frizzled proteins (FZD), LRP5 and LRP6 (representing lipoprotein receptor-related protein 5/6), which will induce inactivation of “destruction complex”, leaving the β-catenin unphosphorylated, also called active β-catenin. Subsequently, active β-catenin will be translocated into the nucleus, where it binds to T cell factor/lymphoid enhancer factor (TCF/LEF) transcription factors. TCF/LEF/β-catenin will further trigger Wnt-dependent pro-fibrotic gene expression, such as TGF-β1/SMAD signaling, RAS, Snail1, Twist1, MMP-7, TRPC6, PAI-1, fibroblasts and also macrophages activation [20]. Non-canonical Wnt/PCP and Wnt/Ca^2+^ pathways are less studied but also considered important, mostly for intercellular interaction and epithelial to mesenchymal cell transition [19]. Polycystin mutation, a known cause of cystic kidney disease, was recognized as a Wnt co-receptor which activates the Wnt/Ca^2+^ pathway [21]. Additionally, Wnt/PCP is considered a factor involved in polycystic kidney disease development through gene mutations such as CELSR1, CELSR2, CELSR3 (representing cadherin EGF LAG seven-pass G-type receptor 1/2/3), VANGL1 and VANGL2 (representing Vang-like protein 1/2). Wnt ligands are divided into two groups. Thereby, Wnt 1, 2, 3, 8a, 8b, 10a and 10b are considered canonical Wnt ligands, whereas Wnt 4, 5a, 5b, 6, 7a, 7b and 11 are recognized as non-canonical Wnt ligands [22]. Currently, 19 Wnt ligands are known, and 16 of them are expressed in renal response to injury [23].

### 4.2. Snail Pathway

Snail is a transcription factor involved in cell cycle arrest, fatty acid metabolism and inflammatory response from kidney injury [24]. It is one of the main factors responsible for EMT processes from proximal tubular epithelial cells (PTECs) and parietal epithelial cells (PECs) involved in renal fibrosis [25]. It is secreted by the Wnt/β-catenin pathway after activating TCF/LEF transcription factors and TGF-β1, or more exactly by SMAD3 transcription factor [26]. The inactivation of GSK3β through Wnt/β catenin activation is involved in Snail activation because GSK3β is responsible for Snail proteolysis [27]. High levels of Snail1 transcription factor were evidenced not only in CKD from immunoglobulin A nephropathy (IgAN) [24], polycystic kidney disease [28] or diabetic nephropathy [24] but also in AKI subjects [29]. Some researchers consider that Snail activation represents a breaking point in AKI transition to CKD; therefore, it is identified as a promising therapeutic target [30].

### 4.3. MMP-7 Signaling

Another transcriptional target of Wnt/β catenin signaling is MMP-7, also named Matrilysin. Matrilysin is a calcium- and zinc-dependent endopeptidase involved in collagen IV, laminin and E-cadherin proteolysis [31]. It also promotes FMT [32], cell apoptosis [32], EMT [33] and podocyte destruction through Nephrin damage [33]. Between Wnt/β catenin and MMP-7, there is a bilateral interrelation because MMP-7 can also control the Wnt/β catenin pathway through a feedback mechanism [34]. Despite a lot of evidence of renal fibrosis being triggered by high MMP-7 activity, some researchers found that MMP-7 is renoprotective in different AKI models. Therefore, additional studies are needed on MMP-7 to evaluate its role in renal fibrosis involvement [35].

### 4.4. PAI-1 Signaling

PAI-1 contains a binding site for the TCF/LEF complex, which is the reason why it is considered an end result of the Wnt/β catenin pathway [36]. PAI-1 is a serine protease inhibitor and contributes to renal fibrosis via negative actions through urokinase-type plasminogen activator (uPA) and tissue-type plasminogen activator (tPA) with subsequently low intracellular plasmin. A low level of plasmin induces low fibrin degradation and ECM accumulation with fibrosis [37]. According to Rabieian et al., factors that promote hypoxia and oxidative stress raise PAI-1 activity [38]. Recently, Yao et al. evidenced lower interstitial fibrosis and downregulation of FMT in PAI-1-depleting models [39].

### 4.5. TRPC6

TRPC6 is another important transcriptional target of Wnt/β-catenin. Through its role in intrapodocyte calcium influx, TRPC6 is responsible for cytoskeleton destabilization, mitochondrial damage and cell apoptosis. Thereby, TRPC6 over-activation is considered a mediator of podocyte damage with subsequent focal and segmental glomerulosclerosis (FSGS). In animal models, the TRPC6 knockout gene evidenced lower interstitial fibrosis and α-SMA expression [40].

### 4.6. RAS Activation

Over the last few years, RAS has been one of the most important pro-fibrotic studied factors, with numerous encouraging results. Recent bioinformatics analyses discovered that RAS genes present TCF/LEF binding sites, which suggests that RAS is controlled by Wnt/β-catenin. The hypothesis was proved through a β-catenin inhibitor (ICG-001), used on mouse models, which evidenced abolished RAS activation [41]. RAS is divided into classical and alternative pathways. The classical RAS pathway starts with renin secretion from juxtaglomerular cells. Renin serum will further convert liver-formed angiotensinogen into angiotensin I (ANG I). Furthermore, ANG I, through angiotensinogen converting enzyme (ACE), will form ANG II. Over the last years, the discovery of pro renin receptors (PRR) established that the binding of prorenin (a renin precursor) or renin itself to this receptor (PRR) can induce a three-to-five-fold increase in renin activity. Apart from the regulation of RAS, PRR is also involved in the activation of different intracellular transcriptional factors, such as Wnt, mitogen-activated protein kinases (MAP), nuclear factor kappa-light-chain-enhancer of activated B cells (NFkB), phosphoinositide 3-kinase (PI3K) and oxidative stress [42]. Moreover, other researchers sustain PRR involvement in PAI-1, TGF-β1, and high fibronectin production [43]. By binding to angiotensin type 1 receptor (AT1R), the final product of the RAS classical pathway, ANG II, activates Janus kinases and mitogen-activated protein kinases (MAPKs), induce intracellular calcium influx through Ca^2+^ channels, and upregulate TGF-β1/SMAD2 and SMAD3, leading to kidney fibrosis [44]. In addition, ANG II and aldosterone raise the production of ECM proteins, such as fibronectin, collagen I, PAI-1 and PAI-2 [45]. Alternatively, the RAS pathway system acts as an anti-fibrotic factor. It starts with angiotensin (1–9), angiotensin-(1–7) and ends with Almandine. Almandine binds to AT2R, mass receptor (MasR) and member D (MrgD) to create anti-fibrotic pathways [44].

### 4.7. TGF-β1 and SMAD Signaling Pathway

**Figure 2:**
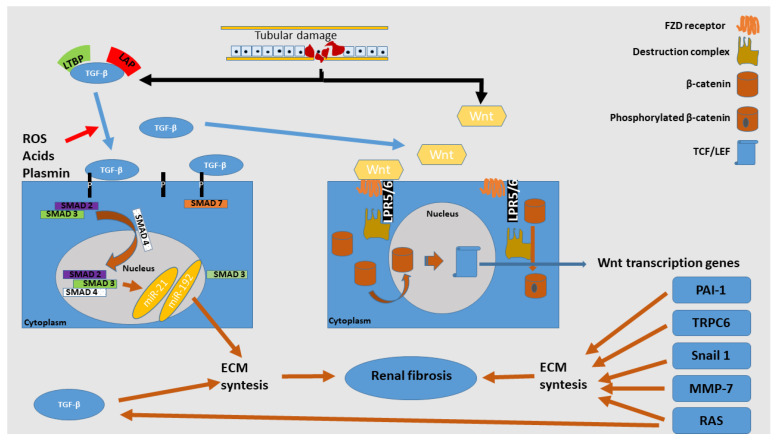
TGF-β1 and Wnt/β-catenin activation in tubular damage. ROS, acids and plasmin can induce TGF-β1 detachment from LTBP and LAP. TGF-β1 induces phosphorylation to the SMAD2/3 complex, which is translocated into the nucleus by SMAD4 and exerts pro-fibrotic transcription genes through different miRNAs. The right part of the picture represents the Wnt/β-catenin pathway. Wnt ligands bind to FZD and LPR5/6, which will induce the inactivation of the “destruction complex”, leaving β-catenin unphosphorylated. Unphosphorylated β-catenin can translocate into the nucleus, bind to TCF/LEF and, subsequently, induce Wnt-dependent pro-fibrotic gene expression. Acronyms: ECM—extracellular matrix; FZD—Frizzled receptor; LAP—latency-associated peptide; LRP5/6—lipoprotein receptor-related protein 5/6; LTBP—latent TGF-β1 binding protein; miR—micro ribonucleic acid; MMP-7—matrix metalloproteinase-7; PAI-1—plasmin activator inhibitor-1; RAS—renin-angiotensin system; ROS—reactive oxygen species; SMAD—acronym for the Caenorhabditis elegans SMA, “small” worm phenotype and MAD family, “mothers against decapentaplegic” of genes in Drosophila; Snail—gene encoded a nuclear protein similar to Drosophila embryonic protein snail; TCF/LEF—T cell factor/lymphoid enhancer factor; TGF-β—transforming growth factor beta; TRPC6—transient receptor potential canonical 6; Wnt—wingless.

TGF-β1 is a powerful mediator responsible for EMT, FMT, cell apoptosis and dedifferentiation of tubular cells [46]. There are three isoforms of TGF, TGF-β1, -β2 and -β3, but TGF-β1 is predominant and can be produced by all kidney resident cells. Meng et al. concluded that TGF-β1 and SMAD signaling are closely connected and together can represent a key pathological mechanism in kidney fibrosis. As a general concept, SMAD signaling represents an equilibrium between SMAD3 and SMAD7 transcription factors, with other factors that act to inhibit or stimulate each of them. SMAD3 is a pro-fibrotic marker with increased activity during fibrogenesis. It is responsible for myofibroblast’s activation and the overproduction of ECM. SMAD3 is upregulated not only from TGFβ-1 but also from C-reactive protein (CRP), ANG II or advanced end products (AGEs) [47]. On the other hand, SMAD7 acts as an anti-fibrotic factor, being the main pathway involved in SMAD3 inhibition. With high TGF-β1 levels, SMAD7 is significantly reduced, and some studies evidenced accelerated fibrosis after kidney injuries in mice with a knockout gene for SMAD7 [48]. TGF-β1 is synthesized in a latent form (TGF-β1 attached to latency-associated peptide (LAP) and latent TGF-β1 binding protein (LTB)), which is present in target tissues. Stimuli, like reactive oxygen species (ROS), acids or plasmin, can release TGF-β1 from LAP and LTBP, resulting in active TGF-β1. This will further bind to type II TGF-β1 receptor (TβRII), then to type I TGF-β1 receptor (TβRI), and further, it will bind to phosphorylate receptor associated-SMADs (R-SMADs), represented by the SMAD2 and SMAD3 complex. To counteract the SMAD3 pro-fibrotic effects, SMAD7 induces TβRI destruction via ubiquitin E3. In pathological states, overactivated SMAD3 inhibits ubiquitin E3 through ligases, such as SMAD ubiquitination regulatory factor 1 (Smurf1), Smurf2 and Arkadia, and subsequently inactivates SMAD7 [47]. Histone methyltransferase (SET9) upregulates SMAD3 and induces high SMA expression in renal fibrosis [49]. GSK3β inhibition induces higher cyclic adenosine monophosphate (cAMP) response element-binding protein (CREB) activity. Higher CREB will bind to CREB-binding protein (CBP), a transcriptional co-activator of SMAD3 activation [50]. Another key protein in inhibition control of SMAD3 synthesis is SMAD2. Through some experiments with the SMAD3 knockout gene, a lower renal fibrosis level in diabetic [46], obstructive [51] and hypertensive nephropathy [52] were evidenced. SMAD2 binds to SMAD3 to form an oligomeric complex that will further activate SMAD4, responsible for the cytoplasm to nuclear cell translocation of the complex previously mentioned. SMAD4 knockout gene evidenced attenuated renal fibrosis in UUO, apparently through SMAD3 inhibition [53]. SMAD3 promotes macrophage infiltration through its direct effect on macrophage chemotactic protein-1 (MCP-1) [54]. The end result of the TGF-β1/SMAD signaling pathway activates different noncoding ribonucleic acids (RNAs), which include miRNAs (representing microRNAs), piwi-interacting RNAs, siRNAs (representing small interfering RNAs) and lncRNAs (representing long noncoding RNAs). MiRNAs are small-length RNAs of approximately 22 nucleotides and, like other RNAs, are in control of different gene expressions. TGF-β1/SMAD signaling can induce miR-21 [55], miR-192 [56] or miR-377 and inhibits the expression of miR-200 and miR-29 [47]. In vitro studies evidenced FMTs abolished by miR-21 knockout genes, and in vivo studies revealed a lower level of kidney fibrosis in obstructive nephropathy in mouse models [57]. MiR-192 evidenced a strong association with higher tubulointerstitial fibrosis in IgAN individuals [58]. Liu et al. evidenced lower levels of kidney ischemia-reperfusion injury (IRI) with abolished inflammation and oxidative stress through miR-377 inhibition [59]. Exosome-encapsulated miR-29 (Exo/miR-29), administrated by intramuscular injection on mouse models, evidenced attenuated renal fibrosis through direct TGF-β1 inhibition [60]. AT-rich interactive domain 2-IR (Arid2-IR) is another lncRNA regulated via SMAD signaling. It is responsible for cell cycle protection control. Active SMAD3 inhibits Arid2-IR with subsequent cell cycle arrest in the G1 phase. SMAD inhibition evidenced higher levels of Arid2-IR, associated with increased cell regeneration and decreased apoptosis [61]. Growth arrest specific-5 (GAS5) is a lncRNA inhibited by the SMAD pathway. In vitro studies with GAS5 knockout showed blocked renal fibrosis [62]. In addition, Lnc-TSI (representing TGF-β/SMAD3-interacting long noncoding RNA), correlated with the renal fibrosis severity, has an anti-fibrotic effect and is downregulated via SMAD signaling. The klotho molecule is secreted by tubular convoluted segments and promotes anti-fibrotic properties through TGF-β1 inhibition and phosphate clearance. Klotho is recognized as an anti-aging protein [63]. These entire pathways are summarized in Figure 2.

### 4.8. Cell Death Pathways

There are three important cell death pathways that are considered to be heavily involved in inflammatory responses, starting from dying cells with subsequent activation of pro-fibrotic pathways. Apoptosis is a form of cell death induced by a high exposure to phosphatidylserine (PtdSer) without cell plasma membrane rupture, which will serve as signals for macrophage phagocytosis ending with a “silent” immunological response [64]. The genetical deletion of the apoptosis key protein, such as Fas-associated protein or caspase-8, failed to show implications in AKI murine models. In contrast, necroptosis and ferroptosis induce the rupture of the plasma membrane and regenerative signals via pro-inflammatory pathways, including FMT processes [65]. There are more and more studies that show the involvement of ferroptosis in AKI transition to CKD, but its detailed mechanisms are currently unknown. Ferroptosis appears after lipid peroxidation failure. Central mechanisms involved in ferroptosis are glutathione peroxidase 4 (GPX4), responsible for lipids oxidation of inactive alcohols and intracellular pool glutathione (GSH). Unlike apoptosis and necroptosis, higher levels of oxidized phosphatidylinositol, PtdSer and phosphatidylethanolamine [66] are released through ferroptosis. These will induce redox imbalance with downregulation of T and B cell activation and also inhibition of T cell cross-priming, allowing ferroptotic cells to regenerate via pro-inflammatory mechanisms, which will generate a persistent pro-inflammatory state [65]. Necroptosis is a process that starts with the release of damage-associated molecular patterns (DAMPs) and is controlled through phosphorylation of RIPK1, RIPK2, RIPK3 (representing receptor-interacting serine/threonine-protein kinase 1/2/3) and mixed lineage kinase domain-like pseudokinase (MLKL) [67]. Some consider that necroptosis stimulates dendritic cells (DCs) cross-presentation to CD8+ cytotoxic T cells, which activates myofibroblast proliferation [68]. A recent study evidenced lower levels of renal inflammation, tubular cell apoptosis and RIPK-induced necroptosis via SMAD3 inhibition [69]. Other evidence with correlation to different cell death pathways and renal fibrosis is presented in Table 1.

### 4.9. Hypoxia-Inducible Factor Pathways

Hypoxia-inducible factor (HIF) pathway is largely represented by two HIF1α subunits (HIF1α and HIF2α) and HIF1β. In normal conditions, HIF-1α is fragmented by proteasome intervention after specific prolyl hydroxylase domain (PHD) hydroxylation and Hippel-Lindau protein (pVHL)-E3-ubiquitin ligase binding. In hypoxic environments, PHDs are inhibited, and HIF-1/2α translocates into the cell nucleus to bind HIF-1β, which can induce more than 300 gene transcription, including vascular endothelial growth factor (VEGF), erythropoietin or glucose transporters like glucose transporter 1 (GLUT1) [108]. Due to some PHD inhibitors with subsequent upregulation of HIF-1/2α factors, it was revealed a reduced renal injury through lower macrophage infiltration, apoptosis and vascular cell adhesion molecule 1 (VCAM1) expression [109]. MiR-668, a transcriptional factor induced via HIF activation, promotes renoprotective effects via the downregulation of apoptosis and mitochondrial fragmentation [93]. If some authors consider that HIF factors are renoprotective, others sustain that HIF is implicated in promoting renal fibrosis via TGF-β, neurogenic locus notch homolog protein (Notch), nuclear factor kappa-light-chain-enhancer of activated B cells (NF-κB) or PI3K/Akt (representing phosphatidylinositol 3-kinase/protein kinase B) signaling pathways, regulation of different transcriptional pro-fibrotic genes and also its potential implication in EMT processes [110].

### 4.10. VEGF

The data regarding VEGFs role in kidney fibrosis are contradictory. Some authors indicated that the upregulation of podocyte-derived VEGF induced collapsing glomerulopathy, and, in contrast, the downregulation of VEGF was associated with thrombotic microangiopathy [94,96]. Xu et al. highlighted that the upregulation of VEGF via the activation of HIFα subunits exerts renoprotective effects. Moreover, it was discovered that under HIF activation, miR-21 is upregulated and exerts a protective effect in kidney injury due to its inhibiting role for Thrombospondin-1 [85]. SAR131675 (representing selective VEGF receptor-3 tyrosine kinase inhibitor), used to control lymphangiogenesis, evidenced reduced oxidative stress, apoptosis and lower renal fibrosis [98]. Chen et al. noticed that VEGF is upregulated in nasopharyngeal carcinoma, and it was associated with the upregulation of matrix-metalloproteinases, key elements for EMT processes [97].

### 4.11. Mitochondrial Dysfunction

Recent studies highlighted the importance of key mechanisms in the early elimination of injured mitochondria from damaged kidney tubular cells. Mitochondria homeostasis is performed through three processes: mitochondrial dynamics, mitochondrial mitophagy and mitochondrial biogenesis. Mitochondrial dynamics are represented by mitochondrial fission processes controlled by mitofusin 1 (MFN1), MFN2, OPA1 (a gene encoding a dynamin-like mitochondrial GTPase involved in autosomal dominant optic atrophy) and fusion processes regulated by: mitochondrial dynamics, mitochondrial mitophagy and mitochondrial biogenesis. Mitochondrial dynamics are represented by mitochondrial fission processes controlled by mitofusin 1 (MFN1), MFN2, OPA1 (a gene encoding a dynamin-like mitochondrial GTPase involved in autosomal dominant optic atrophy) and fusion processes regulated by dynamic-related protein 1 (DRP1) [111]. Mitophagy is a selective pathway represented by the autophagy of injured mitochondria. Mitophagy is controlled via the Parkin RBR E2 ubiquitin protein ligase (PINK1-PARK2 pathway, BCL2 interacting protein3-like (BNIP3-NIX) pathway and FUN14 domain containing 1 (FOUNDC1) pathway [111]. Li et al. evidenced that DRP1 knockout genes from PTECs ameliorate apoptosis, inflammation, kidney injury and kidney fibrosis in mouse models [90]. Studies with PINK1/PARK2 evidenced renoprotective effects in AKI subjects caused by ROS and damaged mitochondria removal, relieved inflammatory response, lower tubular epithelial cells apoptosis and Drp1 inhibition [88,89]. Feng et al. concluded that STE20-like kinase 1 (Mst1) genetic ablation induces lower tubular epithelial cells (TECs) damage via inhibiting AMPK signaling and reduced OPA1 expression [91]. A reduced mitophagy with accumulation of ROS, damaged mitochondria and upregulation of inflammatory renal response following IRI mouse models was revealed using the BNIP3 knockout gene [92].

### 4.12. G2/M Arrest Pathway

When PTEC injury is not fully restored, cells undergo a senescent state via cell cycle G2/M arrest, which leads to the activation of specific pro-fibrotic factors [112]. Recent studies evidenced that G2/M-arrested cells form the target of rapamycin (TOR)-autophagy special coupling compartments (TASCCs). Knockout of TASCC gene ameliorated renal fibrosis in mice with CKD. Moreover, Cyclin G1 (CG1) inhibition downregulated TASCC, considered a potential target of renal fibrosis [99]. Researchers evidenced higher activation of Toll-like receptor (TLR) and interleukin 1 (IL-1R) receptors immediately after G2/M cell cycle arrest occurs [113]. Knockout for Myd88, an TLR/IL-1 downstream protein and an NF-κB upstream protein, revealed ameliorated renal fibrosis in kidney damage [100]. Cyclin-dependent kinase inhibitors (CDKIs), such as p16, p21, p27 or p38, are also involved in TECs cell cycle arrest and subsequent death via SMAD3 activation. CDKIs are also incriminated in renal ageing [72]. A hypoxic environment induces increased miR-493 transcription, which induces higher G2/M cell cycle arrest through stathmin (STMN-1) protein inhibition [86].

### 4.13. Innate and Adaptive Immunological Pathways

Renal injuries activate innate immune pathways, such as complement, macrophages or neutrophils and adaptive pathways, represented by lymphocytes.

Some recent studies revealed that complement C1R, C3a/C3aR [114] and C5a/C5aR [115] are involved in tubulointerstitial fibrosis. Moreover, C5aR knockout showed reduced tubulointerstitial fibrosis in diabetic nephropathy [101]. Another primordial element in IRI mouse induction was the activation of innate immune receptors, like TLRs and nod-like receptors (NLRs). These receptors are involved in DAMPs and pathogen-associated molecular patterns (PAMPs) recognition, which will trigger pathways such as MAPK, c-Jun N-terminal kinases (JNK) or NF-κB, leading up to a proinflammatory cascade of chemokines and cytokines [116]. Silva et al. showed that in order to induce renal damage in post-AKI mouse models, the presence of TLR4 is needed [107].

The role of dendritic cells in kidney fibrosis was indicated via ACC220 treatment in Adriamycin mice nephropathy. ACC220 inhibits FMS-like tyrosine kinase 3 (Flt3) expressed by dendritic cells and induces the downregulation of inflammatory cytokines, chemokines, IL-6, IL-1β, CCL2, CCL5 and tumor necrosis factor (TNF) α. This suggests that ACC220 might be a potential treatment for renal fibrosis [103].

In 2019, Mehrotra et al. found that Th17 CD4 positive induces the secretion of IL-17, and by inhibiting them using Orai1, a gene for calcium release, kidney fibrosis is ameliorated [102]. Some studies evaluate the role of IL-10, derived from CD4+ T cell activation, in kidney fibrosis pathways implication. Thereby, IL-10 is proven to activate Janus kinase (JaK1) and Tyrosine kinase 2 (Tyk2), which will induce phosphorylation of transcription proteins involved in anti-inflammatory and anti-fibrotic mechanisms, such as STAT1, 3 and 5 [117]. An in vitro study revealed kidney anti-fibrotic effects, with reduced renal proteinuria and inflammation after IL-10 administration [104]. Co-vaccination with IL-10 reduced tubular damage in mouse models with induced systemic lupus erythematosus (SLE) nephropathy [105]. Double negative T cells ameliorated AKI-cisplatin induced via the downregulation of PTECs apoptosis [106].

Vinuesa et al. evidenced, since 2008, that macrophages exert two phenotypes. According to his experiment, macrophage depletion 1 to 3 days after kidney injury induced a severe injury with low tubular regeneration, whereas depletion of macrophages in the late stages of AKI showed reduced tubulointerstitial fibrosis [118]. Macrophages can be M1 type, classically and initially activated, with pro-inflammatory functions and release of TNF-α, IL-1 and IL-6 or M2-type with anti-inflammatory and later activity in kidney restore processes. M2 is also responsible for IL-4 and IL-10 stimulation. M2-type macrophages may be involved in renal fibrosis through MMT or various growth factors [63]. M2-type macrophages are considered a source and recipient of Wnt7b. In renal injuries, M2 activation releases Wnt7b, which activates non-canonical pathways, and Wnt derived from tubular epithelial cells can induce macrophage activation [119]. Macrophage activation is represented by the MMT process described by recruited macrophages from bone marrow, which become myofibroblasts [120]. In kidney injuries, tubular epithelial cell releases canonical Wnt ligands (such as Wnt 3a and 5a), which will induce cyclin D1, IL-4 and TGF-β1 upregulation with subsequent M1- to M2-type (pro-fibrotic) macrophage transition and proliferation [121]. Therefore, macrophages, as known sources of Wnt proteins and responsible for TGF-β1 and tissue inhibitors of metalloproteinases (TIMPs) release with subsequent ECM synthesis and deposition, are considered key inflammatory cells involved in kidney fibrosis [122]. Some studies sustain that MMT represents more than 60% of myofibroblast origin, and also that the TGF-β1/SMAD3 pathway acts as a regulator for this process [120]. Through RNA sequence studies, it was revealed that TGF-β1 induces MMT via the SMAD3-Src-POU4F1 pathway [70,71].

## 5. Therapeutic Potential in Renal Fibrosis

Animal and pilot studies have confirmed the anti-fibrotic properties of many drugs, but so far, none of these treatments have been considered definitely specific in slowing the progression of renal fibrosis.

Lifestyle, environmental and dietary factors can influence renal disease progression. Kim et al. concluded that Western diets, which include pre-packed foods, red meat, high-sugar drinks, processed meat, high-fat dairy products, corn or sweets, promote renal fibrosis [123]. An in vitro study demonstrated that alcohol abuse increased kidney fibrosis through SMAD7 downregulation [124]. Smoking was associated with increased renal fibrosis in autosomal dominant polycystic kidney disease subjects [125]. Obesity is another factor involved in renal damage; therefore, some authors recommend bariatric surgery to improve kidney function [126]. Duan et al. revealed that swimming, in contrast to sedentary behavior, alleviated renal interstitial fibrosis [127].

Pirfenidone, an anti-fibrotic therapy, has been studied in human trials with encouraging results. It acts as a TGF-β1 inhibitor and evidenced a median of 25% improvement in eGFR decline in 21 FSGS patients evaluated for a 12-month period. Pirfenidone’s reported adverse effects were photosensitive dermatitis, dyspepsia and sedation [128]. A more recently studied protein is Fresolimumab, a monoclonal anti-TGF-β1. It was studied in 32 patients with FSGS, in different doses and placebo compared, but despite good tolerability, the results were inconclusive [129]. Quercetin demonstrated therapeutic effects in AKI post cisplatin via SMAD3 inhibition [130]. Isolated from Resina Toxicodendron, GQ5 is a small compound that inhibits SMAD3 and downregulates renal fibrosis without any obvious adverse effect [131]. A metabolite of vitamin A, all-trans retinoic acid (ATRA), demonstrates SMAD3 inhibition with subsequent SMAD7 upregulations and protective properties in diabetic kidney disease [132]. Astaxanthin significantly reduced renal fibrosis through SMAD2, STAT3 (representing signal transducer and activator of transcription 3), Akt, Snail and β-catenin pathways inhibition [133]. Apigenin (API) acts via the co-inhibition of Wnt/β-catenin and uric acid reabsorption. Used in mouse models, API evidenced lower kidney fibrosis [134]. Additionally, prostaglandin E2 (PGE2) inhibits kidney fibrosis by lowering IDO (representing indoleamine-2, 3-dioxygenase) expression. IDO is highly expressed in renal IRI and activates β-catenin pathways [135]. PAI-039 (representing PAI-1 antibody) downregulates the progression of diabetic nephropathy in mouse models. Body weight loss might be an adverse effect of PAI-039 treatment [136]. RAS inhibition therapy includes numerous drugs. Angiotensin 1 receptor blockers (ARBs), such as Candesartan [137], Olmesartan [138] and Losartan [138], were mostly effective in liver fibrosis reduction, subsequently lowering the activity of αSMA, TIMP-1, Col-1 (representing collagen type 1), TGF-β and MMP-2. Enalapril [139], Ramipril [138] and Lisinopril [140] are ACE inhibitors that induce fibrosis reduction in the heart, kidneys and skin. In some cases, these ARBs and ACE inhibitors can associate with low-level hyperkalemia and minimal raises of serum creatinine [137,138,139,140]. Steroid mineralocorticoid receptor antagonists (Spironolactone) and non-steroid mineralocorticoid receptor antagonists (Finerenone), through their anti-fibrotic and anti-inflammatory effects, managed to induce encouraging renal and cardiovascular outcomes, mostly in diabetic patients, but with increased risk of hyperkaliemia [141,142]. Tolvaptan showed a delayed progression of renal cysts and might be a potential drug in patients with polycystic kidney disease. In an extensive study, 38 of 681 subjects presented higher levels of serum alanine aminotransferase; however, this was reversible after stopping the treatment [143]. Steroids in IgA nephropathy reported some renal improvement but with a high risk of death and infections. There are ongoing trials with lower doses of steroids [144]. In vitro studies with A8011 and Compound 21 (AT2R agonists) for the treatment of heart and skin fibrosis reported lower levels of fibrosis, collagen, TGF-β1, TIMP and MMP 1-9 [145]. Aliskiren, a renin inhibitor, showed a reduction in TGF-β1, αSMA and collagen deposition in kidney and heart fibrosis [145]. Li. et al. demonstrated that Fenofibrate, through its role in Nrf2 (representing nuclear factor erythroid 2-related factor 2) upregulation, downregulates GSH and GPX4 and, subsequently, inhibits ferroptosis in diabetic mouse models [78]. Antidiabetics, like Metformin, evidenced lower podocyte destruction, mesangial cells apoptosis and tubulointerstitial cell senescence via AMPK and mammalian target of rapamycin (mTOR) regulation in diabetic nephropathy. It also showed a reduced cyst-growing rate in autosomal dominant polycystic kidney disease (ADPKD) [146]. TRC160334, a PHD inhibitor, induced the upregulation of HIF factors and renoprotective effects in IRI [87]. Regarding VEGF inhibition, a study proved the protective role of Calcium Dobesilate in diabetic nephropathy via VEGF inhibition and, subsequently, the downregulation of PI3K/AKt/mTor signaling [95]. Mesenchymal stem cells (MSCs) are pluripotent cells harvested from the umbilical cord, bone marrow, adipose tissue or other tissues. Studies revealed that the inoculation of MSCs downregulates apoptosis, oxidative stress and renal fibrosis [147,148]. Tetramethylpyrazine (TMP), an active protein from the Chinese herb Ligusticum wallichii, showed encouraging results in a meta-analysis which included 32 studies. According to Li et al., TMP reduced AKI through apoptosis, autophagy, inflammation and oxidative stress inhibition using NF-κB, TGF-β1/SMAD signaling and MMPs inhibition [149].

## 6. Discussion

Before the 18th century, renal diseases were unknown. Kidney dysfunctions were first discussed by Bright in 1827 when he discovered some forms of glomerulopathies with renal macroscopic changes. Throughout the next fifty years, his work was continued by Frerichs, Langhans and Klebs through different studies on primary glomerular lesions. The 20th century came with tremendous knowledge covering urine formation, sodium retention in edematous states, RAS system, nephrotic syndrome, various pathways of renal disease investigations, tubular-interstitial syndromes, progress in histology and kidney immunology, antihypertensive medication or development of renal replacement therapy and kidney transplant [150]. In 1873, the term “fibrosis” was first described by Beale upon finding an artery with thick walls, which he named “arterio-capillary fibrosis” [151]. In 1894, Hollis WA was the first to define kidney fibrosis after describing macroscopic modification of ischemic kidneys [150]. In the last century, the progress in renal histology and immunology identified many mechanisms involved in kidney fibrosis, mostly activated by ischemic states, inflammatory insults and congenital or acquired disorders of different transcriptional factors involved in collagen synthesis. Considering that most of these pathways were studied in vitro or on animal models, and it is unclear if they can be applied to humans, our work presents an updated overview of the mechanisms involved in AKI transition to CKD, highlighting that renal fibrosis can be prevented and, consequently, decreasing the incidence of CKD in the general population. However, much remains to be done, especially regarding renal fibrosis therapy.

## 7. Conclusions and Future Directions

Nowadays, given the increasing CKD epidemiology, AKI transition to CKD represents a major topic that requires extensive investigation. It is clear that, despite considerable data involving the pro-fibrotic mechanisms and stimuli, the treatment for preventing renal fibrosis is insufficient. As already highlighted, there are numerous pathways incriminated for renal fibrosis, but with little concrete information, and many of them studied only in animal models. TGF-β1, RAS, HIF factors and Wnt/β-catenin mechanisms seem to represent the central pathogenicity in renal fibrosis, but further extensive studies are required to establish the renoprotective role through their inhibition and, additionally, to evaluate them in humans. Currently, the most common therapy in CKD prevention from any kind of AKI is represented by ANG II inhibitors, with indirect effects on TGF-β1s downregulation. Furthermore, even if RAS inhibitors are frequently used in daily practice, most of them are prescribed to delay renal dysfunction in the CKD population and not for the prevention of renal fibrosis in high-risk AKI patients. We look forward to new studies of precise and targeted treatments against proteins, transcriptional factors, signaling pathways, RNAs or other factors involved in kidney fibrosis. Furthermore, new non-invasive screening and assessment tools for renal fibrosis should be explored in order to achieve an early diagnosis.

## Figures and Tables

**Table 1:** Main factors implicated in renal fibrosis.

Class	Name	Pathways	Action	Evidence
**Wnt/β-catenin**	**TRPC6** knockout	Wnt/β-catenin downstream signaling	Ameliorates renal fibrosis and α-SMA expression	[40]
	**Snail1** inhibition by Eucalyptol		Loweres α-SMA expression in tubulointerstitial space	[26]
	**Snail** upregulation		Seen in largest cysts from polycystic renal disease	[28]
	**PAI-1** knockout		Ameliorates tubulointerstitial fibrosis via FMT inhibition and lower collagen I deposition	[37]
	**MMP-7** knockout		Protects from podocyte destruction	[33]
	**MMP-7** upregulation		Promotes apoptosis and FMT	[32]
**ANG II**	**ANG II** upregulation	JAK and MAPK activation Increased intracellular Ca^2+^ influx Upregulation of TGF-β/SMAD2/SMAD3 signaling	Renal fibrosis	[44]
	**Oxymatrine**	ANG II and aldosterone inhibition	Overproduction of fibronectin, collagen I, PAI-1 and PAI-2	[45]
**TGF-β1/SMAD signaling**	**POU4F1** silencing	SMAD3 downstream signaling	Prevents MMT	[70]
	**Src** inhibition		Prevents MMT	[71]
	**SET9** activation		Increases SMA expression	[49]
	**SMAD4** inhibition		Lowers renal fibrosis in UUO mouse models	[53]
	**SMAD3** inhibition		Lowers renal fibrosis in diabetic, obstructive and hypertensive nephropathy	[46,51,52]
	**GSK3β** inhibition		Induces higher CREB activity with lower CBP, essential in SMAD3 activation	[50]
	Snail1 knockout		Reduces renal fibrosis in mice obstructive nephropathy	[29]
	**CDKIs**: p16, p21, p27, p38		Induces tubular epithelial cell death, through G1 cell cycle arrest and are involved in kidney ageing	[72]
	**PAI-1** activation	Klotho inhibition with TGF-β and p53 upregulation	Promotes kidney fibrosis	[73]
	**CRP**	Smad activation via CD32b-ERK/p38 MAP kinase crosstalk pathway	Promotes kidney inflammation and fibrosis	[74]
	**miR-21** knockout	SMAD3 downstream signaling Through PTEN/Akt pathway	In vivo studies—abolished FMT In vitro studies—alleviates renal fibrosis in obstructive nephropathy	[57]
	**miR-192**	SMAD3 downstream signaling	High serum/intrarenal and urinary levels were associated with higher grades of tubulointerstitial fibrosis	[58]
	**Exo/miR-29**	SMAD3 downstream signaling	MiR-29 exerts anti-fibrotic effects through TGF-β1 inhibition	[60]
	**Erbb4-IR**	SMAD dependent lncRNA	Promotes renal fibrosis in diabetic and obstructive nephropathy Erbb4-IR inhibition alleviates renal fibrosis via miR-29 upregulation	[75]
	**Arid2-IR**		Protective in cell cycle control	[61]
	**GAS5**		Inhibits TGF-β1 and is downregulated by SMAD signaling Evidences blocked renal fibrosis in vitro studies	[62]
**Cell death pathways**	**Fn-14** knockout gene	TWEAK pathway	Reduces death of tubular epithelial cells	[76]
	**Pannexin-1** inhibition	MAPK/ERK pathway	Inhibits ferroptosis and evidences decreased serum creatinine, cell necrosis and melondialdehyde expression	[77]
	** Ferrostatin-1 **	Ferroptosis inhibitor	Lowers tissue renal damage in mouse models with cisplatin-induced AKI	[78]
	** VDR **	Inhibits GPX4—key control of ferroptosis	Knockout for VDR in mice evidenced worsened renal injury Paricalcitol activates VDR and evidenced lower AKI stage after cisplatin treatment	[79]
	** XJB-5-131 **	Inhibits ferroptosis	Decreases AKI stage, attenuated inflammation and promoted tubular epithelial cell proliferation	[80]
	** Tocilizumab **	IL-6 and ferroptosis inhibition	Alleviates renal injuries in obstructive nephropathy	[81]
	** Legumain **	Downregulate GPX4—key control of ferroptosis	Alleviates AKI stage in rats	[82]
	**miR-387a-3p** knockout	Ferroptosis transcription factor	Downregulates IRI on mouse models	[83]
	**miR-182-5p** knockout	Ferroptosis transcription factor	Downregulates IRI on mouse models	[83]
	**Necrostatin-1**	RIPK1 inhibition	Ameliorates IRI via HIF-1α/mir-26a/TRPC6/PARP1 inhibition	[84]
	**hsa-miR-500a-3P** knockout (mRNAs for **MLKL**)	SMAD3 downstream signaling	Alleviates kidney injury by necroptosis inhibition	[67]
	**RIPK** upregulation	SMAD3 downstream signaling	Promotes necroptosis	[69]
**Hypoxia pathways**	**HIF-1/2α** stimulation	miR-21 upregulation and VEGF upregulation	Renoprotective due to increased angiogenesis	[85]
	**miR-493** overexpression	STMN-1 inhibition	Is stimulated by hypoxia and induces renal fibrosis through G2/M cell cycle arrest	[86]
	**TRC160334**	PHD inhibitor	Induce HIF-1*α* upregulation with reduced IRI	[87]
	**PINK1/PARK2**	Upregulated mitophagy	Inhibits Drp1, renal inflammation and tubular epithelial cell apoptosis	[88,89]
	**Drp1** knockout	Mitochondrial fragmentation	Ameliorates renal inflammation, renal injury and renal fibrosis	[90]
	**Mst1** knockout	Upregulates mitophagy	Renoprotective via AMPK and OPA1 downregulation	[91]
	**BNIP3** knockout	Downregulates mitophagy	Increased ROS, damaged mitochondria and inflammatory renal response	[92]
	**miR-668**	HIF-1α downstream signaling	Renoprotective due to reduced apoptosis and mitochondrial fragmentation	[93]
	**VEGF** inhibition		Induces thrombotic microangiopathy	[94,95]
	**VEGF** upregulation		Induces collapsing glomerulopathies	[96]
	**VEGF** upregulation		Increases EMT via upregulation of matrix metalloproteinases	[97]
	**SAR131675**	VEGF receptor—tyrosine kinase inhibition	Reduces apoptosis, lymphangiogenesis, inflammation and renal fibrosis	[98]
**G2/M cell cycle arrest**	**GC1** inhibition	TASCC inhibition	Ameliorates renal fibrosis	[99]
	**MYD88** knockout	TLR/IL-1R downstream signaling and NF-κB upstream signaling	Ameliorates renal fibrosis	[100]
**Immunological pathways**	**NOX-D21**	C5a/C5aR inhibition	Attenuates tubulointerstitial fibrosis in diabetic nephropathy	[101]
	**Orai1** knockout	Th17 inhibition	Alleviates renal fibrosis	[102]
	**Flt3** inhibitor	DCs downstream signaling	Reduces proinflammatory cytokines and chemokines, TNF-α, IL-6 and IL-1β	[103]
	**IL-10**	Cd4+ T cell activation	Reduces renal fibrosis in diabetic nephropathy	[104]
	**IL-10** co-vaccination		Reduces tubular damage in SLE nephropathy on mouse models	[105]
	**Double negative T cells** upregulation		PTECs-reduced apoptosis	[106]
	**TLR4** depletion		Diminishes renal damage after Cisplatin induced AKI	[107]

Acronyms: AKI—acute kidney injury; AMPK—AMP (adenosine monophosphate)-activated protein kinase; ANG II—angiotensin II; Arid2-IR—AT-rich interactive domain 2-IR; BNIP3—BCL2 (B-cell lymphoma 2) interacting protein 3; Ca^2+^—calcium; CBP—CREB-binding protein; CDKIs—cyclin-dependent kinase inhibitors; CREB—cyclic adenosine monophosphate response element-binding protein; CRP—C-reactive protein; Erbb4-IR—erb-b2 receptor tyrosine kinase 4-IR; DCs—dendritic cells; DRP1—dynamin-related protein 1; EMT—epithelial to mesenchymal transition; ERK—extracellular-signal-regulated kinase; Exo/miR-29—exosome-encapsulated miR-29; FLT3—Fms-like tyrosine kinase 3; FMT—fibroblast to myofibroblast transition; Fn-14—fibroblast growth factor-inducible 14; GAS5—growth arrest specific-5; GC1—mitochondrial glutamate carrier-1; GPX4—glutathione peroxidase 4; GSK3β—glycogen synthase kinase 3β; HIF—hypoxia-inducible factor; IL—interleukin; IRI—ischemia-reperfusion injury; JAK—Janus kinase; MAP—mitogen-activated protein kinases; MAPK—mitogen-activated protein kinase; MLKL—mixed lineage kinase domain-like pseudokinase; MMP-7—matrix metalloproteinase-7; MMT—macrophage (bone marrow derived) myofibroblast transition; Mst1—macrophage-stimulating 1; NF-κB—nuclear factor kappa-light-chain-enhancer of activated B cells; NOX-D21—crystallographic structures of an active Spiegelmer; OPA1—a gene encoding a dynamin-like mitochondrial GTPase, involved in autosomal dominant optic atrophy; Orai1—calcium release-activated calcium channel protein 1; PAI—plasmin activator inhibitor; PARP1—poly (ADP-ribose) polymerase 1; PHD—prolyl hydroxylase domain; PINK1—PTEN-induced kinase 1; POU4F1—POU domain, class four, transcription factor 1; PTECs—proximal tubular epithelial cells; PTEN/Akt pathway—phosphatase and tensin homolog deleted on chromosome 10/protein kinase B pathway; ROS—reactive oxygen species; RIPK—receptor-interacting protein kinase; SAR131675—selective VEGF receptor-3 tyrosine kinase inhibitor; SET9—histone methyltransferase; SLE—systemic lupus erythematosus; SMA—smooth muscle actin; SMAD—acronym for the Caenorhabditis elegans SMA, “small” worm phenotype and MAD family, “mothers against decapentaplegic” of genes in Drosophila; Snail—gene encoded a nuclear protein similar to Drosophila embryonic protein snail; Src—sarcoma gene; TASCC—TOR (target of rapamycin)-autophagy spatial coupling compartment; TGF-β—transforming growth factor beta; TNF-α—tumor necrosis factor alpha; TLR—Toll-like receptor; TRPC6—transient receptor potential canonical 6; TWEAK—TNF-like weak inducer of apoptosis; UUO—unilateral ureteral obstruction; VDR—vitamin D receptor; VEGF—vascular endothelial growth factor; Wnt/β-catenin—wingless/β-catenin.

## Data Availability

Available at request.

## References

[1] Takaori K., Nakamura J., Yamamoto S., Nakata H., Sato Y., Takase M., Nameta M., Yamamoto T., Economides A.N., Kohno K. (2016). Severity and Frequency of Proximal Tubule Injury Determines Renal Prognosis. J. Am. Soc. Nephrol..

[2] Hewitson T.D., Boon W.C., Simpson E.R., Smith E.R., Samuel C.S. (2016). Estrogens do not protect, but androgens exacerbate, collagen accumulation in the female mouse kidney after ureteric obstruction. Life Sci..

[3] Fiorentino M., Grandalino G., Gesualdo L., Castellano G. (2018). Acute Kidney Injury to Chronic Kidney Disease Transition. Contrib. Nephrol..

[4] (2012). KDIGO Clinical Practice Guideline for Acute Kidney Injury. Kidney Int. Suppl..

[5] Susantitaphong P., Cruz D.N., Cerda J., Abulfaraj M., Alqahtani F., Koulouridis I., Jaber B.L. (2013). Acute Kidney Injury Advisory Group of the American Society of Nephrology. World incidence of AKI: A meta-analysis. Clin. J. Am. Soc. Nephrol..

[6] Mammen C., Al Abbas A., Skippen P., Nadel H., Levine D., Collet J.P., Matsell D.G. (2012). Long-term risk of CKD in children surviving episodes of acute kidney injury in the intensive care unit: A prospective cohort study. Am. J. Kidney Dis..

[7] Ishani A., Xue J.L., Himmelfarb J., Eggers P.W., Kimmel P.L., Molitoris B.A., Collins A.J. (2009). Acute kidney injury increases risk of ESRD among elderly. J. Am. Soc. Nephrol..

[8] Horne K.L., Packington R., Monaghan J., Reilly T., Selby N.M. (2017). Three-year outcomes after acute kidney injury: Results of a prospective parallel group cohort study. BMJ Open.

[9] Chawla L.S., Eggers P.W., Star R.A., Kimmel P.L. (2014). Acute kidney injury and chronic kidney disease as interconnected syndromes. N. Engl. J. Med..

[10] Meng X.M., Nikolic-Paterson D.J., Lan H.Y. (2014). Inflammatory processes in renal fibrosis. Nat. Rev. Nephrol..

[11] Hong K.M., Belperio J.A., Keane M.P., Burdick M.D., Strieter R.M. (2007). Differentiation of human circulating fibrocytes as mediated by transforming growth factor-beta and peroxisome proliferator-activated receptor gamma. J. Biol. Chem..

[12] Lin T.C., Hung L.Y., Chen Y.C., Lo W.C., Lin C.H., Tam K.W., Wu M.Y. (2019). Effects of febuxostat on renal function in patients with chronic kidney disease: A systematic review and meta-analysis. Medicine.

[13] Allison S.J. (2013). Fibrosis: The source of myofibroblasts in kidney fibrosis. Nat. Rev. Nephrol..

[14] Nikolic-Paterson D.J., Wang S., Lan H.Y. (2014). Macrophages promote renal fibrosis through direct and indirect mechanisms. Kidney Int. Suppl..

[15] Boor P. (2012). EP4: A new piece in the fibrotic puzzle. Kidney Int..

[16] Huang P., Yan R., Zhang X., Wang L., Ke X., Qu Y. (2019). Activating Wnt/β-catenin signaling pathway for disease therapy: Challenges and opportunities. Pharmacol. Ther..

[17] Xie H., Miao N., Xu D., Zhou Z., Ni J., Yin F., Wang Y., Cheng Q., Chen P., Li J. (2021). FoxM1 promotes Wnt/β-catenin pathway activation and renal fibrosis via transcriptionally regulating multi-Wnts expressions. J. Cell. Mol. Med..

[18] Schunk S.J., Floege J., Fliser D., Speer T. (2021). WNT-β-catenin signalling—A versatile player in kidney injury and repair. Nat. Rev. Nephrol..

[19] Hu H.H., Cao G., Wu X.Q., Vaziri N.D., Zhao Y.Y. (2020). Wnt signaling pathway in aging-related tissue fibrosis and therapies. Ageing Res. Rev..

[20] Li S.S., Sun Q., Hua M.R., Suo P., Chen J.R., Yu X.Y., Zhao Y.Y. (2021). Targeting the Wnt/β-Catenin Signaling Pathway as a Potential Therapeutic Strategy in Renal Tubulointerstitial Fibrosis. Front. Pharmacol..

[21] Kim S., Nie H., Nesin V., Tran U., Outeda P., Bai C.X., Keeling J., Maskey D., Watnick T., Wessely O. (2016). The polycystin complex mediates Wnt/Ca(2+) signalling. Nat. Cell Biol..

[22] Acebron S.P., Niehrs C. (2016). β-Catenin-Independent Roles of Wnt/LRP6 Signaling. Trends Cell Biol..

[23] Zhou D., Tan R.J., Fu H., Liu Y. (2016). Wnt/β-catenin signaling in kidney injury and repair: A double-edged sword. Lab. Investig..

[24] Simon-Tillaux N., Hertig A. (2017). Snail and kidney fibrosis. Nephrol. Dial. Transplant..

[25] García de Herreros A., Baulida J. (2012). Cooperation, amplification, and feed-back in epithelial-mesenchymal transition. Biochim. Biophys. Acta..

[26] Kim D.Y., Kang M.K., Park S.H., Lee E.J., Kim Y.H., Oh H., Choi Y.J., Kang Y.H. (2017). Eucalyptol ameliorates Snail1/β-catenin-dependent diabetic disjunction of renal tubular epithelial cells and tubulointerstitial fibrosis. Oncotarget.

[27] Zhang S., Qian G., Zhang Q.Q., Yao Y., Wang D., Chen Z.G., Wang L.J., Chen M., Sun S.Y. (2019). mTORC2 Suppresses GSK3-Dependent Snail Degradation to Positively Regulate Cancer Cell Invasion and Metastasis. Cancer Res..

[28] Togawa H., Nakanishi K., Mukaiyama H., Hama T., Shima Y., Sako M., Miyajima M., Nozu K., Nishii K., Nagao S. (2011). Epithelial-to-mesenchymal transition in cyst lining epithelial cells in an orthologous PCK rat model of autosomal-recessive polycystic kidney disease. Am. J. Physiol. Renal Physiol..

[29] Grande M.T., Sánchez-Laorden B., López-Blau C., De Frutos C.A., Boutet A., Arévalo M., Rowe R.G., Weiss S.J., López-Novoa J.M., Nieto M.A. (2015). Snail1-induced partial epithelial-to-mesenchymal transition drives renal fibrosis in mice and can be targeted to reverse established disease. Nat. Med..

[30] García-Sánchez O., López-Hernández F.J., López-Novoa J.M. (2010). An integrative view on the role of TGF-beta in the progressive tubular deletion associated with chronic kidney disease. Kidney Int..

[31] Wozniak J., Floege J., Ostendorf T., Ludwig A. (2021). Key metalloproteinase-mediated pathways in the kidney. Nat. Rev. Nephrol..

[32] Villa-Morales M., Fernández-Piqueras J. (2012). Targeting the Fas/FasL signaling pathway in cancer therapy. Expert Opin. Ther. Targets.

[33] Tan R.J., Li Y., Rush B.M., Cerqueira D.M., Zhou D., Fu H., Ho J., Beer Stolz D., Liu Y. (2019). Tubular injury triggers podocyte dysfunction by β-catenin-driven release of MMP-7. JCI Insight.

[34] Liu Z., Tan R.J., Liu Y. (2020). The Many Faces of Matrix Metalloproteinase-7 in Kidney Diseases. Biomolecules.

[35] Fu H., Zhou D., Zhu H., Liao J., Lin L., Hong X., Hou F.F., Liu Y. (2019). Matrix metalloproteinase-7 protects against acute kidney injury by priming renal tubules for survival and regeneration. Kidney Int..

[36] Malik S.A., Modarage K., Goggolidou P. (2020). The Role of Wnt Signalling in Chronic Kidney Disease (CKD). Genes.

[37] Flevaris P., Vaughan D. (2017). The Role of Plasminogen Activator Inhibitor Type-1 in Fibrosis. Semin. Thromb. Hemost..

[38] Rabieian R., Boshtam M., Zareei M., Kouhpayeh S., Masoudifar A., Mirzaei H. (2018). Plasminogen Activator Inhibitor Type-1 as a Regulator of Fibrosis. J. Cell. Biochem..

[39] Yao L., Wright M.F., Farmer B.C., Peterson L.S., Khan A.M., Zhong J., Gewin L., Hao C.M., Yang H.C., Fogo A.B. (2019). Fibroblast-specific plasminogen activator inhibitor-1 depletion ameliorates renal interstitial fibrosis after unilateral ureteral obstruction. Nephrol. Dial. Transplant..

[40] Gu L.F., Ge H.T., Zhao L., Wang Y.J., Zhang F., Tang H.T., Cao Z.Y., Yu B.Y., Chai C.Z. (2020). Huangkui Capsule Ameliorates Renal Fibrosis in a Unilateral Ureteral Obstruction Mouse Model Through TRPC6 Dependent Signaling Pathways. Front. Pharmacol..

[41] Zhou L., Li Y., Hao S., Zhou D., Tan R.J., Nie J., Hou F.F., Kahn M., Liu Y. (2015). Multiple genes of the renin-angiotensin system are novel targets of Wnt/β-catenin signaling. J. Am. Soc. Nephrol..

[42] Yang T. (2023). Soluble (Pro)Renin Receptor in Hypertension. Nephron.

[43] Zhou G., Wu J., Gu C., Wang B., Abel E.D., Cheung A.K., Huang Y. (2018). Prorenin independently causes hypertension and renal and cardiac fibrosis in cyp1a1-prorenin transgenic rats. Clin. Sci..

[44] AlQudah M., Hale T.M., Czubryt M.P. (2020). Targeting the renin-angiotensin-aldosterone system in fibrosis. Matrix Biol..

[45] Yang Y., Chen S., Tao L., Gan S., Luo H., Xu Y., Shen X. (2019). Inhibitory Effects of Oxymatrine on Transdifferentiation of Neonatal Rat Cardiac Fibroblasts to Myofibroblasts Induced by Aldosterone via Keap1/Nrf2 Signaling Pathways In Vitro. Med. Sci. Monit..

[46] Meng X.M., Tang P.M., Li J., Lan H.Y. (2015). TGF-β/Smad signaling in renal fibrosis. Front. Physiol..

[47] Wu W., Wang X., Yu X., Lan H.Y. (2022). Smad3 Signatures in Renal Inflammation and Fibrosis. Int. J. Biol. Sci..

[48] Liu G.X., Li Y.Q., Huang X.R., Wei L., Chen H.Y., Shi Y.J., Heuchel R.L., Lan H.Y. (2013). Disruption of Smad7 promotes ANG II-mediated renal inflammation and fibrosis via Sp1-TGF-β/Smad3-NF.κB-dependent mechanisms in mice. PLoS ONE.

[49] Shuttleworth V.G., Gaughan L., Nawafa L., Mooney C.A., Cobb S.L., Sheerin N.S., Logan I.R. (2018). The methyltransferase SET9 regulates TGFB1 activation of renal fibroblasts via interaction with SMAD3. J. Cell Sci..

[50] Chen B., Wang P., Liang X., Jiang C., Ge Y., Dworkin L.D., Gong R. (2021). Permissive effect of GSK3β on profibrogenic plasticity of renal tubular cells in progressive chronic kidney disease. Cell Death Dis..

[51] Sato M., Muragaki Y., Saika S., Roberts A.B., Ooshima A. (2003). Targeted disruption of TGF-beta1/Smad3 signaling protects against renal tubulointerstitial fibrosis induced by unilateral ureteral obstruction. J. Clin. Investig..

[52] Kriegel A.J., Liu Y., Fang Y., Ding X., Liang M. (2012). The miR-29 family: Genomics, cell biology, and relevance to renal and cardiovascular injury. Physiol. Genom..

[53] Meng X.M., Huang X.R., Xiao J., Chung A.C., Qin W., Chen H.Y., Lan H.Y. (2012). Disruption of Smad4 impairs TGF-β/Smad3 and Smad7 transcriptional regulation during renal inflammation and fibrosis in vivo and in vitro. Kidney Int..

[54] Zhang F., Tsai S., Kato K., Yamanouchi D., Wang C., Rafii S., Liu B., Kent K.C. (2009). Transforming growth factor-beta promotes recruitment of bone marrow cells and bone marrow-derived mesenchymal stem cells through stimulation of MCP-1 production in vascular smooth muscle cells. J. Biol. Chem..

[55] Zhong X., Chung A.C., Chen H.Y., Meng X.M., Lan H.Y. (2011). Smad3-mediated upregulation of miR-21 promotes renal fibrosis. J. Am. Soc. Nephrol..

[56] Li Y., Zhang J., Shi J., Liu K., Wang X., Jia Y., He T., Shen K., Wang Y., Liu J. (2021). Exosomes derived from human adipose mesenchymal stem cells attenuate hypertrophic scar fibrosis by miR-192-5p/IL-17RA/Smad axis. Stem Cell Res. Ther..

[57] Zhao S., Li W., Yu W., Rao T., Li H., Ruan Y., Yuan R., Li C., Ning J., Li S. (2021). Exosomal miR-21 from tubular cells contributes to renal fibrosis by activating fibroblasts via targeting PTEN in obstructed kidneys. Theranostics.

[58] Fan Q., Lu R., Zhu M., Yan Y., Guo X., Qian Y., Zhang L., Dai H., Ni Z., Gu L. (2019). Serum miR-192 Is Related to Tubulointerstitial Lesion and Short-Term Disease Progression in IgA Nephropathy. Nephron.

[59] Liu Z., Yang Q., Wei Q., Chang Y., Qu M., Yu L. (2019). The protective effect of miR-377 inhibitor against renal ischemia-reperfusion injury through inhibition of inflammation and oxidative stress via a VEGF-dependent mechanism in mice. Mol. Immunol..

[60] Wang H., Wang B., Zhang A., Hassounah F., Seow Y., Wood M., Ma F., Klein J.D., Price S.R., Wang X.H. (2019). Exosome-Mediated miR-29 Transfer Reduces Muscle Atrophy and Kidney Fibrosis in Mice. Mol. Ther..

[61] Huang J., Lai W., Li M., Li C., Lou T., Peng H., Ye Z. (2022). SIS3 Alleviates Cisplatin-Induced Acute Kidney Injury by Regulating the LncRNA Arid2-IR-Transferrin Receptor Pathway. Kidney Blood Press. Res..

[62] Zhang Y.Y., Tan R.Z., Yu Y., Niu Y.Y., Yu C. (2021). LncRNA GAS5 protects against TGF-β-induced renal fibrosis via the Smad3/miRNA-142-5p axis. Am. J. Physiol. Renal Physiol..

[63] Yang L. (2019). How Acute Kidney Injury Contributes to Renal Fibrosis. Adv. Exp. Med. Biol..

[64] Nagata S., Segawa K. (2021). Sensing and clearance of apoptotic cells. Curr. Opin. Immunol..

[65] Maremonti F., Meyer C., Linkermann A. (2022). Mechanisms and Models of Kidney Tubular Necrosis and Nephron Loss. J. Am. Soc. Nephrol..

[66] Wiernicki B., Dubois H., Tyurina Y.Y., Hassannia B., Bayir H., Kagan V.E., Vandenabeele P., Wullaert A., Vanden B.T. (2020). Excessive phospholipid peroxidation distinguishes ferroptosis from other cell death modes including pyroptosis. Cell Death Dis..

[67] Jiang L., Liu X.Q., Ma Q., Yang Q., Gao L., Li H.D., Wang J.N., Wei B., Wen J., Li J. (2019). hsa-miR-500a-3P alleviates kidney injury by targeting MLKL-mediated necroptosis in renal epithelial cells. FASEB J..

[68] Kuppe C., Ibrahim M.M., Kranz J., Zhang X., Ziegler S., Perales P.J., Jansen J., Reimer K.C., Smith J.R., Dobie R. (2021). Decoding myofibroblast origins in human kidney fibrosis. Nature.

[69] Yang Q., Gao L., Hu X.W., Wang J.N., Zhang Y., Dong Y.H., Lan H.Y., Meng X.M. (2021). Smad3-Targeted Therapy Protects against Cisplatin-Induced AKI by Attenuating Programmed Cell Death and Inflammation via a NOX4-Dependent Mechanism. Kidney Dis..

[70] Tang P.M., Zhang Y.Y., Xiao J., Tang P.C., Chung J.Y., Li J., Xue V.W., Huang X.R., Chong C.C., Ng C.F. (2020). Neural transcription factor Pou4f1 promotes renal fibrosis via macrophage-myofibroblast transition. Proc. Natl. Acad. Sci. USA.

[71] Tang P.M., Zhou S., Li C.J., Liao J., Xiao J., Wang Q.M., Lian G.Y., Li J., Huang X.R., To K. (2018). The proto-oncogene tyrosine protein kinase Src is essential for macrophage-myofibroblast transition during renal scarring. Kidney Int..

[72] Fang Y., Gong A.Y., Haller S.T., Dworkin L.D., Liu Z., Gong R. (2020). The ageing kidney: Molecular mechanisms and clinical implications. Ageing Res. Rev..

[73] Gifford C.C., Lian F., Tang J., Costello A., Goldschmeding R., Samarakoon R., Higgins P.J. (2021). PAI-1 induction during kidney injury promotes fibrotic epithelial dysfunction via deregulation of klotho, p53, and TGF-β1-receptor signaling. FASEB J..

[74] You Y.K., Huang X.R., Chen H.Y., Lyu X.F., Liu H.F., Lan H.Y. (2016). C-reactive protein Promotes Diabetic Kidney Disease in db/db Mice via the CD32b-Smad3-mTOR signaling Pathway. Sci. Rep..

[75] Sun S.F., Tang P.M.K., Feng M., Xiao J., Huang X.R., Li P., Ma R.C.W., Lan H.Y. (2018). Novel lncRNA Erbb4-IR Promotes Diabetic Kidney Injury in *db/db* Mice by Targeting miR-29b. Diabetes.

[76] Martin-Sanchez D., Fontecha-Barriuso M., Carrasco S., Sanchez-Niño M.D., Mässenhausen A.V., Linkermann A., Cannata-Ortiz P., Ruiz-Ortega M., Egido J., Ortiz A. (2018). TWEAK and RIPK1 mediate a second wave of cell death during AKI. Proc. Natl. Acad. Sci. USA.

[77] Su L., Jiang X., Yang C., Zhang J., Chen B., Li Y., Yao S., Xie Q., Gomez H., Murugan R. (2019). Pannexin 1 mediates ferroptosis that contributes to renal ischemia/reperfusion injury. J. Biol. Chem..

[78] Li S., Zheng L., Zhang J., Liu X., Wu Z. (2021). Inhibition of ferroptosis by up-regulating Nrf2 delayed the progression of diabetic nephropathy. Free Radic. Biol. Med..

[79] Hu Z., Zhang H., Yi B., Yang S., Liu J., Hu J., Wang J., Cao K., Zhang W. (2020). VDR activation attenuate cisplatin induced AKI by inhibiting ferroptosis. Cell Death Dis..

[80] Zhao Z., Wu J., Xu H., Zhou C., Han B., Zhu H., Hu Z., Ma Z., Ming Z., Yao Y. (2020). XJB-5-131 inhibited ferroptosis in tubular epithelial cells after ischemia-reperfusion injury. Cell Death Dis..

[81] Yang L., Guo J., Yu N., Liu Y., Song H., Niu J., Gu Y. (2020). Tocilizumab mimotope alleviates kidney injury and fibrosis by inhibiting IL-6 signaling and ferroptosis in UUO model. Life Sci..

[82] Chen C., Wang D., Yu Y., Zhao T., Min N., Wu Y., Kang L., Zhao Y., Du L., Zhang M. (2021). Legumain promotes tubular ferroptosis by facilitating chaperone-mediated autophagy of GPX4 in AKI. Cell Death Dis..

[83] Ding C., Ding X., Zheng J., Wang B., Li Y., Xiang H., Dou M., Qiao Y., Tian P., Xue W. (2020). miR-182-5p and miR-378a-3p regulate ferroptosis in I/R-induced renal injury. Cell Death Dis..

[84] Shen B., Mei M., Pu Y., Zhang H., Liu H., Tang M., Pan Q., He Y., Wu X., Zhao H. (2019). Necrostatin-1 Attenuates Renal Ischemia and Reperfusion Injury via Meditation of HIF-1α/mir-26a/TRPC6/PARP1 Signaling. Mol. Ther. Nucleic Acids.

[85] Xu X., Song N., Zhang X., Jiao X., Hu J., Liang M., Teng J., Ding X. (2017). Renal Protection Mediated by Hypoxia Inducible Factor-1α Depends on Proangiogenesis Function of miR-21 by Targeting Thrombospondin 1. Transplantation.

[86] Liu T., Liu L., Liu M., Du R., Dang Y., Bai M., Zhang L., Ma F., Yang X., Ning X. (2019). MicroRNA-493 targets STMN-1 and promotes hypoxia-induced epithelial cell cycle arrest in G_2_/M and renal fibrosis. FASEB J..

[87] Jamadarkhana P., Chaudhary A., Chhipa L., Dubey A., Mohanan A., Gupta R., Deshpande S. (2012). Treatment with a novel hypoxia-inducible factor hydroxylase inhibitor (TRC160334) ameliorates ischemic acute kidney injury. Am. J. Nephrol..

[88] Zhou L., Zhang L., Zhang Y., Yu X., Sun X., Zhu T., Li X., Liang W., Han Y., Qin C. (2019). PINK1 Deficiency Ameliorates Cisplatin-Induced Acute Kidney Injury in Rats. Front. Physiol..

[89] Livingston M.J., Wang J., Zhou J., Wu G., Ganley I.G., Hill J.A., Yin X.M., Dong Z. (2019). Clearance of damaged mitochondria via mitophagy is important to the protective effect of ischemic preconditioning in kidneys. Autophagy.

[90] Li N., Wang H., Jiang C., Zhang M. (2018). Renal ischemia/reperfusion-induced mitophagy protects against renal dysfunction via Drp1-dependent-pathway. Exp. Cell Res..

[91] Feng J., Li H., Zhang Y., Wang Q., Zhao S., Meng P., Li J. (2018). Mammalian STE20-Like Kinase 1 Deletion Alleviates Renal Ischaemia-Reperfusion Injury via Modulating Mitophagy and the AMPK-YAP Signalling Pathway. Cell. Physiol. Biochem..

[92] Tang C., Han H., Liu Z., Liu Y., Yin L., Cai J., He L., Liu Y., Chen G., Zhang Z. (2019). Activation of BNIP3-mediated mitophagy protects against renal ischemia-reperfusion injury. Cell Death Dis..

[93] Wei Q., Sun H., Song S., Liu Y., Liu P., Livingston M.J., Wang J., Liang M., Mi Q.S., Huo Y. (2018). MicroRNA-668 represses MTP18 to preserve mitochondrial dynamics in ischemic acute kidney injury. J. Clin. Investig..

[94] Eremina V., Jefferson J.A., Kowalewska J., Hochster H., Haas M., Weisstuch J., Richardson C., Kopp J.B., Kabir M.G., Backx P.H. (2008). VEGF inhibition and renal thrombotic microangiopathy. N. Engl. J. Med..

[95] Wang Y., Lu Y.H., Tang C., Xue M., Li X.Y., Chang Y.P., Cheng Y., Li T., Yu X.C., Sun B. (2019). Calcium Dobesilate Restores Autophagy by Inhibiting the VEGF/PI3K/AKT/mTOR Signaling Pathway. Front. Pharmacol..

[96] Eremina V., Sood M., Haigh J., Nagy A., Lajoie G., Ferrara N., Gerber H.P., Kikkawa Y., Miner J.H., Quaggin S.E. (2003). Glomerular-specific alterations of VEGF-A expression lead to distinct congenital and acquired renal diseases. J. Clin. Investig..

[97] Chen L., Lin G., Chen K., Liang R., Wan F., Zhang C., Tian G., Zhu X. (2020). VEGF promotes migration and invasion by regulating EMT and MMPs in nasopharyngeal carcinoma. J. Cancer.

[98] Hwang S.D., Song J.H., Kim Y., Lim J.H., Kim M.Y., Kim E.N., Hong Y.A., Chung S., Choi B.S., Kim Y.S. (2019). Inhibition of lymphatic proliferation by the selective VEGFR-3 inhibitor SAR131675 ameliorates diabetic nephropathy in db/db mice. Cell Death Dis..

[99] Canaud G., Brooks C.R., Kishi S., Taguchi K., Nishimura K., Magassa S., Scott A., Hsiao L.L., Ichimura T., Terzi F. (2019). Cyclin G1 and TASCC regulate kidney epithelial cell G_2_-M arrest and fibrotic maladaptive repair. Sci. Transl. Med..

[100] Lemos D.R., McMurdo M., Karaca G., Wilflingseder J., Leaf I.A., Gupta N., Miyoshi T., Susa K., Johnson B.G., Soliman K. (2018). Interleukin-1*β* Activates a MYC-Dependent Metabolic Switch in Kidney Stromal Cells Necessary for Progressive Tubulointerstitial Fibrosis. J. Am. Soc. Nephrol..

[101] Yiu W.H., Li R.X., Wong D.W.L., Wu H.J., Chan K.W., Chan L.Y.Y., Leung J.C.K., Lai K.N., Sacks S.H., Zhou W. (2018). Complement C5a inhibition moderates lipid metabolism and reduces tubulointerstitial fibrosis in diabetic nephropathy. Nephrol. Dial. Transplant..

[102] Mehrotra P., Sturek M., Neyra J.A., Basile D.P. (2019). Calcium channel Orai1 promotes lymphocyte IL-17 expression and progressive kidney injury. J. Clin. Investig..

[103] Wang R., Chen T., Wang C., Zhang Z., Wang X.M., Li Q., Lee V.W.S., Wang Y.M., Zheng G., Alexander S.I. (2019). Flt3 inhibition alleviates chronic kidney disease by suppressing CD103+ dendritic cell-mediated T cell activation. Nephrol. Dial. Transplant..

[104] Fan X., Zhang X., Liu L.C., Kim A.Y., Curley S.P., Chen X., Dworkin L.D., Cooper C.J., Gupta R. (2022). Interleukin-10 attenuates renal injury after myocardial infarction in diabetes. J. Investig. Med..

[105] Martín M.B.T., Satoh M., Hernández P.R., Martínez G.E.A., Petri M.H., Sandoval-García F., Pizano-Martinez O., García-Iglesias T., Corona-Meraz F.I., Vázquez D.M.M. (2021). The DNA co-vaccination using Sm antigen and IL-10 as prophylactic experimental therapy ameliorates nephritis in a model of lupus induced by pristane. PLoS ONE.

[106] Gong J., Noel S., Hsu J., Bush E.L., Arend L.J., Sadasivam M., Lee S.A., Kurzhagen J.T., Hamad A.R.A., Rabb H. (2020). TCR^+^CD4^−^CD8^−^ (double negative) T cells protect from cisplatin-induced renal epithelial cell apoptosis and acute kidney injury. Am. J. Physiol. Renal Physiol..

[107] Andrade S.M., Cenedeze M.A., Perandini L.A., Felizardo R.J.F., Watanabe I.K.M., Agudelo J.S.H., Castoldi A., Gonçalves G.M., Origassa C.S.T., Semedo P. (2018). TLR2 and TLR4 play opposite role in autophagy associated with cisplatin-induced acute kidney injury. Clin. Sci..

[108] Wang Z., Zhang C. (2022). From AKI to CKD: Maladaptive Repair and the Underlying Mechanisms. Int. J. Mol. Sci..

[109] Shu S., Wang Y., Zheng M., Liu Z., Cai J., Tang C., Dong Z. (2019). Hypoxia and Hypoxia-Inducible Factors in Kidney Injury and Repair. Cells.

[110] Liu J., Wei Q., Guo C., Dong G., Liu Y., Tang C., Dong Z. (2017). Hypoxia, HIF, and Associated Signaling Networks in Chronic Kidney Disease. Int. J. Mol. Sci..

[111] Wang Y., Cai J., Tang C., Dong Z. (2020). Mitophagy in Acute Kidney Injury and Kidney Repair. Cells.

[112] Koyano T., Namba M., Kobayashi T., Nakakuni K., Nakano D., Fukushima M., Nishiyama A., Matsuyama M. (2019). The p21 dependent G2 arrest of the cell cycle in epithelial tubular cells links to the early stage of renal fibrosis. Sci. Rep..

[113] Yu S.M., Bonventre J.V. (2020). Acute kidney injury and maladaptive tubular repair leading to renal fibrosis. Curr. Opin. Nephrol. Hypertens..

[114] Peng Q., Li K., Smyth L.A., Xing G., Wang N., Meader L., Lu B., Sacks S.H., Zhou W. (2012). C3a and C5a Promote Renal Ischemia-Reperfusion Injury. J. Am. Soc. Nephrol..

[115] Peng Q., Wu W., Wu K.Y., Cao B., Qiang C., Li K., Sacks S.H., Zhou W. (2019). The C5a/C5aR1 axis promotes progression of renal tubulointerstitial fibrosis in a mouse model of renal ischemia/reperfusion injury. Kidney Int..

[116] Tang P.C., Zhang Y.Y., Chan M.K., Lam W.W., Chung J.Y., Kang W., To K.F., Lan H.Y., Tang P.M. (2020). The Emerging Role of Innate Immunity in Chronic Kidney Diseases. Int. J. Mol. Sci..

[117] Wei W., Zhao Y., Zhang Y., Jin H., Shou S. (2022). The role of IL-10 in kidney disease. Int. Immunopharmacol..

[118] Vinuesa E., Hotter G., Jung M., Herrero F.I., Torras J., Sola A. (2008). Macrophage involvement in the kidney repair phase after ischaemia/reperfusion injury. J. Pathol..

[119] Lu X., Rudemiller N.P., Ren J., Wen Y., Yang B., Griffiths R., Privratsky J.R., Madan B., Virshup D.M., Crowley S.D. (2019). Opposing actions of renal tubular- and myeloid-derived porcupine in obstruction-induced kidney fibrosis. Kidney Int..

[120] Tang P.M., Nikolic P.D.J., Lan H.Y. (2019). Macrophages: Versatile players in renal inflammation and fibrosis. Nat. Rev. Nephrol..

[121] Feng Y., Ren J., Gui Y., Wei W., Shu B., Lu Q., Xue X., Sun X., He W., Yang J. (2018). Wnt/*β*-Catenin-Promoted Macrophage Alternative Activation Contributes to Kidney Fibrosis. J. Am. Soc. Nephrol..

[122] Yang Y., Feng X., Liu X., Wang Y., Hu M., Cao Q., Zhang Z., Zhao L., Zhang J., Guo R. (2019). Fate alteration of bone marrow-derived macrophages ameliorates kidney fibrosis in murine model of unilateral ureteral obstruction. Nephrol. Dial. Transplant..

[123] Kim Y.C., Ganguly S., Nespoux J., Freeman B., Zhang H., Brenner D., Dhar D., Vallon V. (2020). Western diet promotes renal injury, inflammation, and fibrosis in a murine model of Alström syndrome. Nephron.

[124] Yang Q., Chen H.Y., Wang J.N., Han H.Q., Jiang L., Wu W.F., Wei B., Gao L., Ma Q.Y., Liu X.Q. (2020). Alcohol promotes renal fibrosis by activating Nox2/4-mediated DNA methylation of Smad7. Clin. Sci..

[125] Sousa M.V., Amaral A.G., Freitas J.A., Murata G.M., Watanabe E.H., Balbo B.E., Tavares M.D., Hortegal R.A., Rocon C., Souza L.E. (2021). Smoking accelerates renal cystic disease and worsens cardiac phenotype in Pkd1-deficient mice. Sci. Rep..

[126] Sandino J., Martín-Taboada M., Medina-Gómez G., Vila-Bedmar R., Morales E. (2022). Novel Insights in the Physiopathology and Management of Obesity-Related Kidney Disease. Nutrients.

[127] Duan Y.C., Shi L., Jin Z., Hu M., Huang H., Yan T., Zhang K.R. (2021). Swimming Exercise Ameliorates Hypertension-Induced Kidney Dysfunction via Alleviating Renal Interstitial Fibrosis and Apoptosis. Kidney Blood Press. Res..

[128] Cho M.E., Smith D.C., Branton M.H., Penzak S.R., Kopp J.B. (2005). Pirfenidone slows renal function decline in patients with focal segmental glomerulosclerosis. Clin. J. Am. Soc. Nephrol..

[129] Vincenti F., Fervenza F.C., Campbell K.N., Diaz M., Gesualdo L., Nelson P., Praga M., Radhakrishnan J., Sellin L., Singh A. (2017). A Phase 2, Double-Blind, Placebo-Controlled, Randomized Study of Fresolimumab in Patients With Steroid-Resistant Primary Focal Segmental Glomerulosclerosis. Kidney Int. Rep..

[130] Tan R.Z., Wang C., Deng C., Zhong X., Yan Y., Luo Y., Lan H.Y., He T., Wang L. (2020). Quercetin protects against cisplatin-induced acute kidney injury by inhibiting Mincle/Syk/NF-κB signaling maintained macrophage inflammation. Phytother. Res..

[131] Ai J., Nie J., He J., Guo Q., Li M., Lei Y., Liu Y., Zhou Z., Zhu F., Liang M. (2015). GQ5 Hinders Renal Fibrosis in Obstructive Nephropathy by Selectively Inhibiting TGF-β-Induced Smad3 Phosphorylation. J. Am. Soc. Nephrol..

[132] Sierra M.E., Rodríguez R.R., Namorado T.C., Molina E., Romero T.D., Pedraza C.J., Reyes J.L. (2019). All-Trans Retinoic Acid Attenuates Fibrotic Processes by Downregulating TGF-β1/Smad3 in Early Diabetic Nephropathy. Biomolecules.

[133] Diao W., Chen W., Cao W., Yuan H., Ji H., Wang T., Chen W., Zhu X., Zhou H., Guo H. (2019). Astaxanthin protects against renal fibrosis through inhibiting myofibroblast activation and promoting CD8^+^ T cell recruitment. Biochim. Biophys. Acta. Gen. Subj..

[134] Li Y., Zhao Z., Luo J., Jiang Y., Li L., Chen Y., Zhang L., Huang Q., Cao Y., Zhou P. (2021). Apigenin ameliorates hyperuricemic nephropathy by inhibiting URAT1 and GLUT9 and relieving renal fibrosis via the Wnt/β-catenin pathway. Phytomedicine.

[135] Pan B., Zhang H., Hong Y., Ma M., Wan X., Cao C. (2021). Indoleamine-2,3-Dioxygenase Activates Wnt/β-Catenin Inducing Kidney Fibrosis after Acute Kidney Injury. Gerontology.

[136] Liu Y., Wang L., Luo M., Chen N., Deng X., He J., Zhang L., Luo P., Wu J. (2019). Inhibition of PAI-1 attenuates perirenal fat inflammation and the associated nephropathy in high-fat diet-induced obese mice. Am. J. Physiol. Endocrinol. Metab..

[137] Kim M.Y., Cho M.Y., Baik S.K., Jeong P.H., Suk K.T., Jang Y.O., Yea C.J., Kim J.W., Kim H.S., Kwon S.O. (2012). Beneficial effects of candesartan, an angiotensin-blocking agent, on compensated alcoholic liver fibrosis—A randomized open-label controlled study. Liver Int..

[138] Zhu Q., Li N., Li F., Zhou Z., Han Q., Lv Y., Sang J., Liu Z. (2016). Therapeutic effect of renin angiotensin system inhibitors on liver fibrosis. J. Renin Angiotensin Aldosterone Syst..

[139] Tan W.Q., Fang Q.Q., Shen X.Z., Giani J.F., Zhao T.V., Shi P., Zhang L.Y., Khan Z., Li Y., Li L. (2018). Angiotensin-converting enzyme inhibitor works as a scar formation inhibitor by down-regulating Smad and TGF-β-activated kinase 1 (TAK1) pathways in mice. Br. J. Pharmacol..

[140] Brilla C.G., Funck R.C., Rupp H. (2000). Lisinopril-mediated regression of myocardial fibrosis in patients with hypertensive heart disease. Circulation.

[141] Bomback A.S., Kshirsagar A.V., Amamoo M.A., Klemmer P.J. (2008). Change in proteinuria after adding aldosterone blockers to ACE inhibitors or angiotensin receptor blockers in CKD: A systematic review. Am. J. Kidney Dis..

[142] Bakris G.L., Agarwal R., Anker S.D., Pitt B., Ruilope L.M., Rossing P., Kolkhof P., Nowack C., Schloemer P., Joseph A. (2020). FIDELIO-DKD Investigators. Effect of Finerenone on Chronic Kidney Disease Outcomes in Type 2 Diabetes. N. Engl. J. Med..

[143] Torres V.E., Chapman A.B., Devuyst O., Gansevoort R.T., Perrone R.D., Koch G., Ouyang J., McQuade R.D., Blais J.D., Czerwiec F.S. (2017). Tolvaptan in Later-Stage Autosomal Dominant Polycystic Kidney Disease. N. Engl. J. Med..

[144] Lv J., Zhang H., Wong M.G., Jardine M.J., Hladunewich M., Jha V., Monaghan H., Zhao M., Barbour S., Reich H. (2017). Effect of Oral Methylprednisolone on Clinical Outcomes in Patients With IgA Nephropathy: The TESTING Randomized Clinical Trial. JAMA.

[145] Gross O., Girgert R., Rubel D., Temme J., Theissen S., Müller G.A. (2011). Renal protective effects of aliskiren beyond its antihypertensive property in a mouse model of progressive fibrosis. Am. J. Hypertens..

[146] Fang Y., Li F., Qi C., Mao X., Wang F., Zhao Z., Chen J.K., Zhang Z., Wu H. (2020). Metformin effectively treats *Tsc1* deletion-caused kidney pathology by upregulating AMPK phosphorylation. Cell Death Discov..

[147] Nargesi A.A., Lerman L.O., Eirin A. (2017). Mesenchymal Stem Cell-derived Extracellular Vesicles for Renal Repair. Curr. Gene Ther..

[148] Birtwistle L., Chen X.M., Pollock C. (2021). Mesenchymal Stem Cell-Derived Extracellular Vesicles to the Rescue of Renal Injury. Int. J. Mol. Sci..

[149] Li J., Gong X. (2022). Tetramethylpyrazine: An Active Ingredient of Chinese Herbal Medicine With Therapeutic Potential in Acute Kidney Injury and Renal Fibrosis. Front. Pharmacol..

[150] Reubi F. (1987). On the history of kidney disease. Schweiz. Med. Wochenschr..

[151] Beale L.S. (1873). Arterio-Capillary Fibrosis. Br. Med. J..

